# Effect of Combined DASH Diet with Sodium Restriction on Renal Function

**DOI:** 10.34067/KID.0000000000000427

**Published:** 2024-04-25

**Authors:** Kentaro Kohagura

**Affiliations:** Dialysis Unit, University of the Ryukyus Hospital, Nishihara-cho, Okinawa, Japan

**Keywords:** GFR, glomerular hyperfiltration, hypertension

BP control is crucial for preventing the progression of CKD. Dietary interventions controlling sodium and potassium intake play an important role in the management of hypertension. The Dietary Approach to High Blood Pressure (DASH) is a high-potassium diet that is indicated to be effective in controlling BP^1^ and in preventing the progression of CKD.^2^ The DASH-Sodium trial has demonstrated that the results obtained by the combination of low-sodium intake and the DASH diet on BP were superior to the results of either intervention alone.^1^ Although the augmented effect of the DASH diet and sodium restriction on BP implies that the DASH low-sodium diet might also be beneficial for slowing the progression of CKD, the effect of this intervention on the eGFR remains to be determined.

In the current issue of *Kidney360*, Morales-Alvarez *et al.* examined the effects of the combination of sodium intake and DASH diet on eGFR, as well as that of the either alone, using the cystatin C formula in patients with prehypertension and stage 1 hypertension.^3^ This study is a part of the DASH-Sodium trial. This study demonstrated that while a short course of DASH diet does not affect eGFR compared with the control diet, a low-sodium diet, when combined with the DASH diet, resulted in a significant reduction in the eGFR compared with a high-sodium diet. The advantages of the DASH-Sodium trial were that the dietary intake was controlled and compliance was confirmed by monitoring urinary sodium and potassium levels. In addition, the influence of behavioral factors and dietary compliance was minimized, making it possible to study the exact biological effects of each diet on eGFR. Cystatin C–based assessment of eGFR was another positive aspect of this study.

This study demonstrated that the DASH diet by itself did not affect eGFR. This seems appropriate because the previous study had shown that the DASH diet alone caused a modest reduction in BP compared with its combination with a low-sodium diet,^3^ thereby justifying its minimal effect on glomerular hemodynamics. Meanwhile, the DASH low-sodium diet caused the lowest absolute systolic and diastolic BP in a previously conducted study,^1^ which resulted in a significant reduction in eGFR of −3.41 ml/min per 1.73 m^2^ compared with the DASH high-sodium diet.^3^ The effect on eGFR decline was attenuated by adjustment for diastolic BP and 24-hour urinary potassium excretion, suggesting that its effect is partly mediated by changes in diastolic BP and potassium load. On the pressure–diuretic curve, DASH diet did not effect on the x-intercept, but significantly increased the slope, indicating the involvement of natriuresis in its hypotensive mechanism.^4^ These findings indicate that the glomerular hemodynamics is affected by the significant reduction in BP by enhanced natriuresis effects of the DASH low-sodium diet. Under normal conditions, an autoregulation system in the afferent arteriole is responsible for maintaining glomerular hemodynamics over a wide range of BP changes. Tubuloglomerular feedback (TGF) is one of the major autoregulatory mechanisms responsible for maintaining glomerular pressure and single nephron GFR. However, its degree of feedback response varies depending on the systemic volume status.^5^ In the setting of a low-sodium diet, in which the renin-angiotensin system is generally activated, GFR could be reduced because of enhanced TGF-associated vasoconstriction.^5^ Such resetting of TGF may partly account for the reduced eGFR during DASH low-sodium diet observed in this study.

The patients in this study were mainly middle-aged, and their renal function was within the normal range.^3^ The adaptation of TGF response to a low-sodium diet would be favorable for maintenance of volume status and systemic BP by prevention of further natriuresis. However, we need to be cautious about the long-term effects of the DASH low-sodium diet on eGFR. A study conducted by Rule *et al.* demonstrated that approximately 40% of healthy adults in their 50s had pathological findings of vascular luminal narrowing due to arteriosclerosis,^6^ and in such patients, a decrease in eGFR could reflect glomerular hypoperfusion, which can induce renal ischemia. In a subanalysis of the African-American Study of Kidney Disease and Hypertension conducted on Black patients with hypertensive nephrosclerosis, tight BP control was significantly associated with lower eGFR in both the short and long terms in patients with proteinuria ≤0.22 g/gCr.^7^ Therefore, whether an acute decrease in eGFR is beneficial or detrimental to the subsequent course of eGFR might depend on the underlying pathophysiology of the patients with hypertension. Because recent studies did not include information on albuminuria, it is difficult to conclude the consistency of these results with the results of clinical trials in which renoprotective interventions such as renin-angiotensin system inhibitors^8^ caused an acute decline in renal function in patients with CKD with albuminuria, followed by a slowing of the decline.

The DASH diet emphasizes vegetables, fruits, whole grains, and lean proteins and limits foods high in salt, added sugars, and saturated fats. It is high in potassium, calcium, and magnesium. The DASH Sodium trial showed the short-term effects of the DASH diet combined with further salt reduction on BP and eGFR.^1,3^ Further studies are needed to determine its long-term effects and feasibility. In addition, the results of this study have two important implications for future research. First, tight BP control has been shown to be beneficial for patients with hypertension, including elderly patients as well for preventing cardiovascular disease, but with a potential risk of incident CKD.^9^ A recent clinical trial involving mostly middle-aged to elderly people demonstrated that sodium restriction was successful in lowering BP, independent of hypertension status and antihypertensive medication.^10^ Future studies need to validate the long-term safety of the DASH low-sodium diet on renal function with and without antihypertensive treatment in patients with hypertension. Second, glomerular hyperfiltration after nephron loss is an important common pathway for the progression of CKD. Future studies need to determine the effect of the DASH low-sodium diet on long-term GFR and renal outcomes in patients with hypertension and CKD with and without albuminuria.

## Supplementary Material
