# Supplementary material for: Effect of Combined DASH Diet with Sodium Restriction on Renal Function
Source: Kidney360. 2024 Apr 25;5(4):487–8. doi: 10.34067/KID.0000000000000427 (PMC11093539; doi:10.34067/KID.0000000000000427)
Supplement: Supplementary file 1 [file kidney360-5-487-s001.pdf]

ASN American Society of Nephrology JASN Journal of the American Society of Nephrology KIDNEY360 Accessing Our World From Every Angle CJASN Clinical Journal of the American Society of Nephrology

ASN Journal Disclosure Form

As per ASN journal policy, I have disclosed any financial relationship or commitment held by myself and/or my spouse/partner in the past 36 months as included below. I have listed my Current Employer below to indicate there is a relationship requiring disclosure. If no relationship exists, my Current Employer is not listed.

K. Kohagura reports the following:
Employer: University of the Ryukyus Hospital; Ownership Interest: Sustain Happiness; Research Funding: Daiichi Sankyo, Kyowa-Hakko-Kirin, Chugai, Teijin Pharma, Torii, Asteras, Sanwa Kagaku Kenkyusho, Ohtsuka, Terumo; Honoraria: Daiichi Sankyo, Kyowa-Hakko-Kirin, Teijin Pharma, Torii, MSD, Asteras, Chugai, Sanwa Kagaku Kenkyusho.Novartis, Mochida, Terumo, Taishyo-Toyama, Phyzer, Ono, Baxter, Beilinger, Kowa,; and Other Interests or Relationships: NPO, Japan Kidney Association.

I understand that the information above will be published within the journal article, if accepted, and that failure to comply and/or to accurately and completely report the potential financial conflicts of interest could lead to the following: 1) Prior to publication, article rejection, or 2) Post-publication, sanctions ranging from, but not limited to, issuing a correction, reporting the inaccurate information to the authors' institution, banning authors from submitting work to ASN journals for varying lengths of time, and/or retraction of the published work.

Name: Kentaro Kohagura
Manuscript ID: K360-2024-000157R1
Manuscript Title: EFFECT OF COMBINED DASH DIET WITH SODIUM RESTRICTION ON RENAL FUNCTION
Date of Completion: March 4, 2024
Disclosure Updated Date: March 4, 2024
